# Leukocytes as risk markers for cardiovascular disease in adolescents: association with birth characteristics, nutritional status and biochemical tests

**DOI:** 10.1016/j.rppede.2015.12.003

**Published:** 2016

**Authors:** Pedro Paulo do Prado, Franciane Rocha de Faria, Eliane Rodrigues de Faria, Sylvia do Carmo Castro Franceschini, Silvia Eloiza Priore

**Affiliations:** a Universidade Federal de Viçosa (UFV), Viçosa, MG, Brazil; b Universidade Federal do Espírito Santo (UFES), Alegre, ES, Brazil

**Keywords:** Leukocyte count, Risk factors, Cardiovascular diseases, Adolescent

## Abstract

**Objective:**

To evaluate the correlation between the number of leukocytes and cardiovascular risks associated with birth characteristics, nutritional status and biochemical tests.

**Methods:**

Cross-sectional study developed with 475 adolescents, born between 1992 and 2001, in the municipality of Viçosa (MG). Maternal medical records were analyzed in the hospital units, and the following was recorded: birth weight and length, head circumference, chest circumference, Apgar score, gestational age. In adolescents, body mass index, skinfold thickness, body composition, blood count, biochemical tests and clinical variables were also assessed. The statistical analyses was carried out using Statistical Package for Social Sciences (SPSS) version 20.0 and Data Analysis and Statistical Software (STATA) with Kruskal–Wallis, Mann–Whitney, chi-square or Fisher's exact tests and Linear Regression. Significance level was set at *α*<0.05. The study was approved by the Research Ethics Committee of UFV for studies with human subjects.

**Results:**

Weight and birth length, head and chest circumference were higher among boys. In adolescents, the number of leukocytes was higher in individuals with excess weight and body fat and high adiposity index, waist-to-height ratio and waist circumference. Only altered triglycerides showed differences between leukocyte medians. Regardless of the anthropometric variable of the final regression model, the stage of adolescence, number of platelets, eosinophils, monocytes and lymphocytes were associated with the increase in leukocytes.

**Conclusions:**

The birth variables were not associated with changes in leukocyte numbers, whereas the anthropometric variables were good indicators for a higher leukocyte count, regardless of the stage of adolescence and gender.

## Introduction

The relationship between cardiovascular disease (CVD) and risk factors in the early stages of life can be evidenced in the literature. Birth weight is related to cardiovascular risk in adolescence.^1^

From prepubertal period, individuals are exposed early to cardiovascular risk factors, which act negatively in the intimae of blood vessels, leading to the onset of atherosclerosis.^2^ Cardiovascular risk factors (CVRF) are defined as modifiable, such as smoking, high total cholesterol, high LDL, low HDL, hypertension, sedentary lifestyle, and obesity; and not modifiable, such as age, family history of CVD, and sex.^3^^,^ 4

Considering the silent development of atherosclerosis and the role of excess weight in childhood and adolescence as CVRF,^2^ the need for early investigation of these factors is emphasized in order to reduce morbidity and mortality rates from CVD in adulthood.^3^

Adolescence is a phase of exposure to several risk factors and many of the habits acquired in this phase are maintained in adulthood, with health consequences.^4^ Exposure to these factors may be associated with the inflammatory process. Because of the association of inflammatory process with cardiovascular risk factors, especially in childhood and adolescence, different inflammatory markers involved in each stage of atheromatous plaque formation have been studied, including leukocytes.^5^ The number of leukocytes is recognized as an inflammatory marker and predictor of cardiovascular events.^6^ Leukocyte subpopulations are related to the inflammatory process.^7^

Knowing the influence of birth conditions in the development of CVD, our objective was to evaluate the relationship of the number of leukocytes with the cardiovascular risks associated with birth characteristics, nutritional status, body composition, and biochemical tests.

## Method

Cross-sectional study developed with adolescents of both sexes, born in Viçosa, MG, from 1992 to 2001. The sample was distributed according to the stages of adolescence, described as follows: early adolescence (10–13 years); middle adolescence (14–16 years); and late adolescence (17–19 years).^8^ The study design was approved by the Institutional Review Board of UFV (Process No. 163/2012).

Sample selection was done using data from the Live Birth Information System (SINASC) through Datasus (Information Technology at the SUS Service), filtered by the mother's place of residence. An average number of births was calculated due to underreporting of records in the system, totaling 12,090 births for the survey period. This total number of births was used as population to establish the sample size using the Epi Info software version 6.04, from a specific formula for cross-sectional studies. Because the primary outcome is multiple CVRF, a prevalence of 50% was used, which provides greater sample size, conservative prevalence,^9^ acceptable variability of 5% and 95% confidence interval. The minimum sample size included 372 adolescents. Because of the expected sample loss due to the search time interval and the possibility of not finding the data of birth, 20% were added to the initial calculation given a number of 447 subjects. The study enrolled 475 adolescents.

From maternal medical records of all hospital births, a database was built containing birth variables, such as weight, length, head and chest circumference, Apgar score, and gestational age. Birth weight was classified as low (<2500g), insufficient (2500–2999g), appropriate (3000–3999g), and large for gestational age (>4000g).^10^ Length and head circumference at birth were classified using the Ministry of Health criteria, with percentiles as reference.^11^

The next step was to find this population during adolescence. The source search was rural and urban, public and private schools. Subjects were included in the study if agreed in participation and if birth data was available, regardless of the stage of adolescence and regarding sex. Otherwise, these were excluded and made up new draw among eligible adolescents. After obtaining written informed consent from those responsible or from the adolescents over 18 years, the subjects were instructed on previous care and days to carry out the biochemical tests, bioelectrical impedance analysis, clinical examination, and nutritional assessment.

Anthropometric (weight, height), body composition, and biochemical data were collected in the Health Division of the Federal University of Viçosa, MG. Subsequently, a return was scheduled to apply the socioeconomic and lifestyle questionnaires, in addition to delivering test results and nutritional assessment.

Weight was measured in electronic digital scale (Kratos^®^), 150kg capacity, 50g sensitivity. To measure height, a portable stadiometer (Alturexata^®^) was used, with 2.13m length and 0.1cm resolution. Body mass index (BMI) was calculated by the ratio of body weight (kg) to height (m^2^), classified in *z*-scores, according to age and sex, using the World Health Organization proposal.^12^ To assess body composition, vertical bioelectrical impedance with eight tactile electrodes (InBody 230^®^) was used. Waist circumference (WC) was measured at midpoint between the lower margin of the last rib and the iliac crest, in duplicate, with inelastic flexible 2m tape measure, divided into centimeters and subdivided into millimeters. After the evaluation, calculation of waist-to-height ratio (WHtR) and body adiposity index (BAI) was done.

In biochemical tests, total cholesterol, lipoprotein high and low density lipoprotein (HDL and LDL), and triglycerides were measured. Lipid profile rating was based on the I Atherosclerosis Prevention Guidelines in Childhood,^13^ which regards as inadequate the borderline and high values. This reference was also used to evaluate fasting insulin and insulin resistance, which was calculated by the mathematical model Homeostasis Model Assessment – Insulin Resistance (HOMA-IR). Fasting blood glucose was evaluated according to the International Committee of Diabetes Mellitus Diagnosis that considers impaired fasting glucose ≥100mg/dL. Complete blood count and uric acid were classified according to sex and age, according to reference values of Bioclin-Quibasa kit (Quibasa Química Básica Ltda., Belo Horizonte, Brazil).

Leukocyte count was performed using the electric impedance method (Coulter T890 instrument), with the Beckman Coulter kit (Beckman Coulter GmbH, Krafeld, Germany) and reference values for up to 12 years (4500–13,500mm^3^), 13–16 years (4500–13,000mm^3^), 17–18 years (4500–12,500mm^3^), and above 18 years (4500–11,500mm^3^).

Blood pressure was measured based on the protocol established by the VI Brazilian Guideline on Hypertension,^14^ with automatic inflation blood pressure monitor Omron^®^ Model HEM-741 CINT (Omron Healthcare Inc., Lake Forest, IL, USA), favored by the Brazilian Society of Cardiology, classified from P90.

For sedentary behavior, the time spent during the week and at the weekend in front of the TV, video game and computer was assessed and characterized as screen time (ST). As a classification criterion, ST≥2h/day was considered as sedentary behavior.^15^

For data analysis, the Statistical Package for Social Sciences (SPSS, IBM, Chicago, IL, USA) version 20.0 was used. The Kolmogorov–Smirnov test was used to determine the normality of the numerical variables. To compare the numerical variables, Kruskal–Wallis test for three or more independent groups and the Mann–Whitney test for two independent groups were used.

The difference between the proportions was assessed using the chi-square and Fisher exact tests, when necessary. The linear regression models were constructed using the Analysis and Statistical Data software (Stata, Stata Corp., College Station, TX, USA). For regression analysis, continuous variables were used. To evaluate the association between leukocyte count and CV risk factors, adjustment for sex and adolescence phase was made. The independent variables included in the regression models had *p*<0.20 in the simple regression. For the analysis of models, the dependent variable, leukocytes, was transformed into logarithm. Because the anthropometric and body composition variables have multicollinearity, five different multiple regression models were generated. The significance level was α<0.05.

## Results

Study participants were 475 adolescents and 238 (50.1%) were female. When stratifying by adolescence phase, 221 (46.5%) in the early phase, 132 (27.8%) in the middle phase, and 122 (25.7%) in the late phase were enrolled.

Many records had missing values, such as missing Apgar score and gestational age, particularly in the early years of the survey. However, it was observed that most adolescents showed Apgar score ≥7 in the first and fifth minutes, 318 (94.6%) and 379 (98.9%) respectively, which implies good fetal vitality. As for gestational age, it was observed that 97.8% of births were full term (37–41 weeks), six (1.9%) preterm (≤36 weeks), and one (0.3%) post-term. Regarding the type of delivery, there were 157 (33.4%) births by vaginal delivery and 313 (66.6%) by cesarean section.

Median birth weight was 3.100g, higher among boys (*p*<0.001), as well as length at birth (*p*=0.007) and head (*p*<0.001) and thoracic (*p*=0.001) circumference. In adolescence, it was observed that female adolescents had higher values of %BF (*p*<0.001); WC (0.02); WHtR (*p*<0.001) and BAI (*p*<0.001) and there was no difference in relation to BMI. The biggest TT was observed among females, compared to males, without significant difference (*p*=0.09) (Table 1).

**Table 1:** Median, minimum and maximum values of birth and adolescence variables in total sample and stratified by sex.

		Total sample			Male			Female		*p* -value
		n (%)	Median (Min–Max)		n (%)	Median (Min–Max)		n (%)	Median (Min–Max)	
*Birth*										
	Weight (g) a	474	3100 (1480–4500)		236 (96.6)	3200 (1900–4500)		238 (100)	3050 (1480–4350)	<0.001 b
	Length (cm) a	466	50 (38–56)		234 (98.7)	50 (41–56)		232 (97.5)	49 (38–53)	0.007 b
	HC (cm) a	471	34 (28–39)		235 (99.2)	34.5 (31–39)		236 (99.2)	34 (28–37)	<0.001 b
	TC (cm) a	470	33 (27–39)		234 (98.7)	33 (29–39)		236 (99.2)	33 (27–37)	0.001 b

*Adolescence*										
	BMI (kg/m^2^)	475	19.46 (13.22–40.05)		237 (100)	19.27 (13.22–40.05)		238 (100)	19.67 (14.04–34.90)	0.10 b
	%BF (%)	475	21 (5.6–48.7)		237 (100)	16.1 (5.6–43.5)		238 (100)	25.5 (9.1–48.7)	<0.001 b
	WC (cm)	475	71 (51.2–118)		237 (100)	69.5 (51.2–118)		238 (100)	71.5 (53.5–100)	0.02 b
	WHtR (cm)	475	0.44 (0.35–0.71)		237 (100)	0.43 (0.35–0.67)		238 (100)	0.45 (0.36–0.71)	<0.001 b
	BAI (%)	475	24.42 (14.19–42.75)		237 (100)	22.07 (14.19–41.25)		238 (100)	25.93 (18.46–42.75)	<0.001 b
	Screen time/h	475	128.57 (34.29–240)		237 (100)	125.71 (42.86–239.29)		238 (100)	132.99 (34.29–240)	0.09 b

*Sedentary behavior*										
	TT≥2h	274 (57.7)	–		108 (45.6)	–		93 (39.1)	–	0.15 c
	TT<2h	201 (42.3)	–		129 (54.4)	–		145 (60.9)	–	

a Missing values.

b Mann–Whitney test.

c Chi-square.

Min, minimum; Max, maximum; BMI, body mass index; %BF, percentage of body fat; WHtR, waist-to-height ratio; BAI, body adiposity index; ST, screen time; HC, head circumference; TC, thoracic circumference; WC, waist circumference.

In this study, there was no difference between the number of leukocytes and birth weight in the total sample (*p*=0.92) or stratified by sex, male (*p*=0.77) or female (*p*=0.43). The same happened in the evaluation of adolescence phase (*p*=0.42). The number of leukocytes was greater in female adolescents compared to male (*p*<0.001) (Table 2).

**Table 2:** Median, minimum and maximum values of the number of leukocytes in relation to birth variables, anthropometric measurements, biochemical tests, and clinical examinations in adolescents.

		Leukocytes		*p* -value
		n (%)	Median (Q1–Q3)	
*Birth weight (g)*				
	<2500	35 (7.4)	5900 (4900–6900)	0.92 *
	2500–2999	121 (25.5)	5600 (4500–6850)	
	3000–3999	308 (64.8)	5700 (4900–6800)	
	≥4000	10 (2.1)	5750 (4250–6575)	

*Length at birth (cm)*				
	<45	7 (1.5)	5900 (4800–6300)	0.97 *
	≥45 to <53	446 (93.9)	5700 (4800–6800)	
	≥53	22 (4.6)	5900 (5000–6400)	

*1st minute Apgar*				
	≤6	18 (5.4)	6050 (5350–7025)	0.13 **
	≥7	318 (94.6)	5600 (4675–6700)	

*5th minute Apgar*				
	≤6	4 (1.1)	5450 (4200–8725)	0.97 **
	≥7	279 (98.9)	5600 (4800–6700)	

*Head circumference (cm)*				
	<32	19 (4.0)	6300 (5800–6900)	0.30 *
	≥32 to <36	361 (76.0)	5700 (4800–6800)	
	≥36	95 (20.0)	5500 (4700–6600)	

*Adolescence phase*				
	Early	221 (46.5)	5600 (4700–6700)	0.42 *
	Middle	132 (27.8)	5700 (4800–6975)	
	Late	122 (25.7)	5900 (4900–6925)	

*Sex*				
	Male	237 (49.9)	5400 (4600–6350)	<0.001 **
	Female	238 (50.1)	6000 (5000–7300)	

*Nutritional status*				
	Low weight a ^,^ b	21 (4.4)	5000 (4250–6350)	
	Eutrophic a	345 (72.6)	5600 (4700–6700)	0.008 *
	Excess weight b	109 (22.9)	6000 (5100–7450)	

*Percentage body fat (%)*				
	Eutrophic	275 (57.9)	5600 (4600–6500)	0.002 **
	Excess fat	200 (42.1)	6000 (4925–7300)	

*BAI (%)*				
	Normal	424 (89.3)	5600 (4700–6700)	0.002 **
	Changed	51 (10.7)	6400 (5200–7700)	

*Waist circumference (cm)*				
	Normal	430 (90.5)	5600 (4700–6700)	<0.001 **
	Changed	45 (9.5)	6300 (5400–8000)	
*WHtR (cm)*				
	Normal	419 (88.2)	5600 (4700–6700)	<0.001 **
	Changed	56 (11.8)	6350 (5400–7950)	

*Total cholesterol (mg/dL)*				
	<150md/dL	196 (41.3)	5700 (4800–6700)	0.93 **
	<150md/dL	279 (58.7)	5600 (4800–6800)	

*Triglycerides (mg/dL)*				
	<100md/dL	408 (85.9)	5600 (4700–6700)	0.001 **
	≥100md/dL	67 (14.1)	6300 (5300–7400)	
*LDL (mg/dL)*				
	<100md/dL	310 (65.3)	5800 (4800–7000)	0.13 **
	≥100md/dL	165 (34.7)	5500 (4850–6500)	

*HDL (mg/dL)*				
	<45md/dL	307 (64.6)	5600 (4700–6800)	0.18 **
	≥45md/dL	168 (35.4)	5900 (4925–6775)	

*Sedentary behavior*				
	TT≥2h	201 (42.3)	5690 (4900–6800)	0.96 **
	TT<2h	274 (57.7)	5700 (4600–6800)	

*Blood pressure (mmHg)*				
	<P90	460 (96.8)	5700 (4800–6800)	0.91 **
	≥P90	15 (3.2)	5800 (5400–6200)	

* Kruskal–Wallis test.

** Mann–Whitney test.

BAI, body adiposity index; WHtR, waist-to-height ratio; ST screen time; LDL, low density lipoprotein; HDL, high density lipoprotein; Q1, first quartile; Q3, third quartile.

a,b Equal letters mean no statistical difference.

In the evaluation of leukocytes in adolescence, higher values were found in individuals with excess weight (*p*=0.004) and body fat (*p*=0.02); BAI above P90 (*p*=0.002); higher values of WHtR (*p*<0.001) and WC (*p*<0.001).

Regarding lipid profile, only triglycerides showed difference between the number and leukocytes. In adolescents with high TG, leukocytes were higher (*p*=0.001).

No statistical differences were found in white blood cell count, regarding sedentary behavior and among adolescents with blood pressure over P90.

After linear regression analysis between the birth, biochemical, clinical, and anthropometric variables, in relation to the number of leukocytes, the final models adjusted for sex and adolescence are shown in Table 3. These models included eosinophils, lymphocytes, monocytes and platelets (*p*<0.001); triglycerides (*p*=0.016); HOMA score (*p*=0.01); insulin (*p*=0.005); head circumference (*p*=0.14); chest circumference (*p*=0.16); BMI (*p*=0.0005); %BF (*p*=0.001); WHiR (*p*<0.001); WC (*p*=0.004), and BAI (*p*<0.001). The five multiple regression models were performed in order to verify greater explanatory power, but it was observed that any of the used models showed an association of anthropometric parameters and body composition with leukocyte count. Regardless of the anthropometric variable used to assess the number of leukocytes in adolescence in relation to the aforementioned variables, with the exception of BAI and %BF, they all presented the same association with adolescence, sex, number of platelets, eosinophils, monocytes, and lymphocytes in the final model. The 01 (BMI) and 05 (WC) models had the best coefficient of determination (*R* ^2^=0.46). After the five models residual plots analysis, it was found that the residues were distributed linearly over the values. This shows that the linear regression models were adequate. It is also noted on standardized residual charts and predicted values, that the first were distributed uniformly around the mean.

**Table 3:** Final Model of linear regression of association between leukocytes, anthropometric, clinical and laboratory variables adjusted for sex and adolescence phase.

Variable	Model 1-BMI ( *R* ^2^ = 0.46)				Model 2-BAI ( *R* ^2^ = 0.45)				Model 3-%BF ( *R* ^2^ = 0.44)		
	*β*	SE	95%CI		*β*	SE	95%CI		*β*	SE	95%CI
BMI (kg/m^2^)	0.010	0.002	0.005–0.015		–	–	–		–	–	–
%BF (%)	–	–	–		–	–	–		0.004	0.001	0.002–0.006
BAI (%)	–	–	–		0.009	0.002	0.004–0.013		–	–	–
WC (cm)	–	–	–		–	–	–		–	–	–
WHtR (cm)	–	–	–		–	–	–		–	–	–
F. Adol.	0.06	0.012	0.039–0.088		0.08	0.012	0.058–0.106		0.086	0.012	0.062–0.109
Sex	0.06	0.018	0.027–0.100		–	–	–		–	–	–
Platelets (mil/mm^3^)	0.0005	0.0001	0.0002–0.0009		0.0006	0.0001	0.0002–0.0009		0.0005	0.0001	0.0002–0.0009
Eosinophils (mm^3^)	0.0002	0.00003	0.0001–0.0002		0.0001	0.00003	0.0001–0.0002		0.0001	0.00003	0.0001–0.0002
Monocytes (mm^3^)	0.0004	0.00008	0.0003–0.0006		0.0005	0.00008	0.0003–0.0006		0.0005	0.00008	0.0003–0.0006
Lymphocytes (mm^3^)	0.0002	0.00001	0.0001–0.0002		0.0002	0.00001	0.0001–0.0002		0.0002	0.00001	0.0001–0.0002

Variables	Model 4-WHtR ( *R* ^2^ = 0.45)							Model 5-WC ( *R* ^2^ = 0.d)			
	*β*		SE		95%CI			β		SE	95%CI
BMI (kg/m^2^)	–		–		–			–		–	–
%BF (%)	–		–		–			–		–	–
BAI (%)	–		–		–			–		–	–
WC (cm)	–		–		–			0.004		0.0009	0.002–0.005
WHtR (cm)	0.56		0.16		0.245–0.887			–		–	–
Adol. P	0.08		0.012		0.056–0.104			0.06		0.012	0.038–0.088
Sex	0.05		0.019		0.016–0.091			0.06		0.018	0.026–0.099
Platelets (mil/mm^3^)	0.0005		0.0001		0.0002–0.0008			0.0005		0.0001	0.0002–0.0009
Eosinophils (mm^3^)	0.0001		0.00003		0.0001–0.0002			0.0002		0.00003	0.0001–0.0002
Monocytes (mm^3^)	0.0004		0.00008		0.0003–0.0006			0.0004		0.00008	0.0003–0.0006
Lymphocytes (mm^3^)	0.0002		0.00001		0.0001–0.0002			0.0002		0.00001	0.0001–0.0002

BMI, body mass index; %BF, percentage of body fat; BAI, body adiposity index; WC, waist circumference; WHtR, waist-to-height ratio; Adol. P, adolescence phase; –, variable not included in the regression model.

## Discussion

This study evaluated the behavior of birth variables, anthropometric, biochemical tests, and clinical examinations in adolescence with the increase in the number of leukocytes, as a biomarker for the risk of CVD.

The study comprised adolescents with leukocyte values within normal range, as the objective was to evaluate this variable relationship with CV risk factors. Regarding birth weight, associations were found; however, in adolescence it can be observed that individuals with higher fat percentage, excess weight, and hypertriglyceridemia had higher counts of this biological marker for inflammation.

Leukocyte count evaluation is related to a subclinical inflammation, and one does not necessarily need to find changed values for long-term effect to be seen. In the inflammatory process, leukocyte activation occurs, which once activated starts the production of other inflammatory markers.

Conditions at birth, especially birth weight, are a factor related to the development of CVD^1^ and may be linked to leukocytes, biological risk factors for these diseases. This fact could not be observed in this study, in which birth weight was not associated with changes in leukocyte count, unlike the population-based study in northern Finland that found association between underweight at birth and increased numbers of leukocytes, particularly in female adolescents. The identification of inflammation association in adolescents may prevent CVD in adulthood.^16^

In this study, no differences in leukocyte numbers were found in relation to adolescence stages. Contrary to this finding, high inflammation levels were associated with leukocytes in adolescents, aged 13–16 years, who participated in a study performed in England and Wales.^17^

Higher leukocyte numbers were seen among female, a finding that supports the work performed with Finnish and American teenagers, in which the girls had higher number of leukocytes.^16^^,^ 18 The value of this finding is related to the greater exposure of women to the inflammatory process due to the pathophysiological consequences of this condition, particularly when associated with oral contraceptive use that is considered an independent risk for systemic inflammation.^16^

The adolescents in the study with changes in anthropometric and body composition variables had higher number of leukocytes. A study performed in Alegre, ES, Brazil, also showed higher leukocyte and lymphocytes values for adolescents with higher WC and %BF values.^19^ Obesity is characterized by chronic inflammation that leads to changes in the immune system and may be associated with type 2 diabetes and CVD.^20^ In this context, leukocytes may serve as biomarkers or even mediator and link obesity, inflammation and insulin resistance.^6^

Regarding lipid profile, only adolescents with increased triglycerides showed differences between leukocyte counts. Knowing the association of triglycerides with increased risk of coronary heart disease^21^ and that leukocytes may be considered biomarkers for CVD,^16^ they can be used in clinical practice of health care for adolescents to identify cardiovascular risks.

The non-association in this study of leukocyte count to CV risk factors, such as high cholesterol and LDL, low HDL, sedentary behavior, and blood pressure >P90, may be related to the homogeneity of the sample with respect to these variables; however, our data are similar to those found by in the Ten Towns Heart Health Study, which also showed no association between leukocytes and traditional cardiovascular risk factors. The importance of identifying inflammation markers early in life is related to the fact that it plays a causative role of association, in adulthood, between leukocytes and CVD.^17^

There was a relationship between platelets and excess weight. The mean platelet volume is considered an indicator of platelet activity; its increase was demonstrated in several acute vascular events and it is associated with obesity.^22^ Increased platelet activation is known to trigger atherosclerosis and plays an important role in its progression. The platelet and leukocyte poll relationship is associated with higher frequency of adverse cardiovascular outcomes.^23^ White blood cells are related to atherosclerosis regardless of risk factors and may be considered as a low cost and easy to interpret marker in the diagnosis of atherosclerosis.^24^

The increase in total concentration of white blood cells is a risk factor, regardless of morbidity and mortality from coronary heart disease, peripheral artery disease, and stroke.^25^ A Spanish study found an association between leukocyte and subpopulations with hypertriglyceridemia and low HDL, as well as for components of metabolic syndrome. Individuals at higher quartiles of leukocytes had increased risk of developing the syndrome (*p*<0.001). This association was also seen for all leukocyte subtypes, except basophils.^26^ These data support the findings of this study in which leukocyte subpopulations remained in all final regression models.

Among the leukocyte subpopulations, monocytes are described as the predominant cell type in the inflammatory profile in atherosclerotic processes. In adult patients with endocrine disorders, monocytes may be predictors of macrovascular complications.^27^

Eosinophils are multifunctional leukocytes involved in the initiation and propagation of inflammatory responses, and therefore have a role in the pathogenesis of inflammatory diseases.^28^ Eosinophils have a compound which binds to the vascular cell adhesion molecule-1 (VCAM-1) on the endothelium and infiltrate after lying in infected or inflamed region and do phagocytosis of small particles mediated by the antigen-ntibody complex.^28^

Lymphocytes are present in the immune response in all phases of atherosclerosis and are relate to inflammatory markers, when analyzed in relation to obese adolescents.^29^ Individuals with excess weight and body fat, in addition to having macrophages infiltrated into the tissue, also have lymphocytes that produce cytokines and contribute positively to local tissue inflammation.^30^

It can be concluded that in this study, the variables of birth were not associated with changes in the number of leukocytes, whereas anthropometric changes in adolescence have shown be good indicators of larger number of leukocytes, regardless of adolescence stage and sex. These variables were predictive for the increase of platelet count and leukocyte subpopulations.

In this study, although we found no association between leukocyte and cardiovascular risk factors, such as sedentary behavior, blood pressure>P90, high cholesterol and LDL, and low HDL, leukocytes and their subpopulations may be considered inexpensive and effective biomarkers for identification of cardiovascular risk in adolescents, as it is a subclinical inflammation. Thus, the assessment of the number of leukocytes can be another test used in clinical practice, as it is associated with the process of atherogenesis in the presence of cardiovascular risk factors and other markers.

This study has limitations due to its own cross-sectional design, which unable the identification and/or interpretation of the temporality of associations found. However, the results obtained can be used in other studies due to its own characteristics, such as sample size, the representativeness of the adolescent population in the municipality, and the similarity of this group with Brazilian adolescents.

Knowing the relationship between changes in leukocyte values and cardiovascular changes and knowing that such processes begin in childhood and adolescence and persist in adulthood, it is essential to identify these adolescents, in order to reduce exposure to CV risk factors.
