# Supplementary material for: Leukocytes as risk markers for cardiovascular disease in adolescents: association with birth characteristics, nutritional status and biochemical tests
Source: Rev Paul Pediatr. 2016 Jan-Mar;34(1):38–46. doi: 10.1016/j.rppede.2015.12.003 (PMC4795720; doi:10.1016/j.rppede.2015.12.003)
Supplement: Translation

# Translation: Leucócitos como marcadores de risco para doenças cardiovasculares na adolescência: associação com características de nascimento, situação nutricional e exames bioquímicos

**Authors:** Pedro Paulo do Prado, Franciane Rocha de Faria, Eliane Rodrigues de Faria, Sylvia do Carmo Castro Franceschini, Silvia Eloiza Priore

**Affiliations:** a Universidade Federal de Viçosa (UFV), Viçosa, MG, Brasil; b Universidade Federal do Espírito Santo (UFES), Alegre, ES, Brasil

**Keywords:** Contagem de leucócitos, Fatores de risco, Doenças cardiovasculares, Adolescente

## Abstract

**Objetivo:**

Avaliar relação do número de leucócitos com riscos cardiovasculares associados às características de nascimento, situação nutricional e exames bioquímicos.

**Métodos:**

Estudo transversal, desenvolvido com 475 adolescentes, nascidos de 1992 a 2001, em Viçosa (MG). Buscaram-se os prontuários maternos nas unidades hospitalares. Foram registrados: peso e comprimento ao nascer, perímetro cefálico, perímetro torácico, boletim de Apgar, tempo de gestação. Na adolescência, avaliaram-se índice de massa corporal, pregas cutâneas, composição corporal, hemograma, exames bioquímicos e variáveis clínicas. Para análise usaram-se Statistical Package for the Social Sciences (SPSS) versão 20.0 e Data Analysis and Statistical Software (Stata), com os testes de Kruskal-Wallis, Mann-Whitney, qui-quadrado ou Exato de Fisher e regressão linear. Nível de significância adotado α<0,05. O estudo foi aprovado pelo Comitê de Ética e Pesquisa com seres humanos da UFV.

**Resultados:**

O peso e o comprimento ao nascer, perímetro cefálico e torácico foram maiores entre os meninos. Na adolescência, o número de leucócitos foi maior nos indivíduos com excesso de peso e gordura corporal e elevados índice de adiposidade corporal, relação cintura-estatura e perímetro da cintura. Apenas os triglicerídeos alterados apresentaram diferença entre as medianas de leucócitos. Independentemente da variável antropométrica do modelo final de regressão, a fase da adolescência, o número de plaquetas, eosinófilos, monócitos e linfócitos associou-se ao aumento de leucócitos.

**Conclusões:**

As variáveis de nascimento não se associaram às alterações nos números de leucócitos, enquanto as variáveis antropométricas mostraram-se bons indicadores para maior número de leucócitos, independentemente da fase da adolescência e sexo.

## Introdução

A relação entre doenças cardiovasculares (DCV) e fatores de risco nos estágios iniciais da vida pode ser evidenciada na literatura. O peso de nascimento está relacionado aos riscos cardiovasculares na adolescência.^1^

Desde o período pré-púbere, os indivíduos estão precocemente expostos a fatores de risco cardiovasculares, que atuam de forma negativa na íntima dos vasos, levam ao início da doença aterosclerótica.^2^ Os fatores de risco cardiovascular (FRCV) são definidos em modificáveis, como tabagismo, colesterol total elevado, LDL elevado, HDL baixo, hipertensão arterial, sedentarismo e obesidade; e não modificáveis, como idade, histórico familiar de DCV e sexo.^3^^,^ 4

Considerando-se o desenvolvimento silencioso da doença aterosclerótica e o papel do excesso de peso na infância e adolescência como FRCV,^2^ destaca-se a necessidade de investigação precoce desses fatores, a fim de reduzir as taxas de morbimortalidade por DCV na vida adulta.^3^

A adolescência é uma fase de exposição a diversos fatores de risco e muitos dos hábitos adquiridos nessa fase são mantidos na vida adulta, com consequências para a saúde.^4^ A exposição a esses fatores pode estar associada ao processo inflamatório. Em função da associação do processo inflamatório aos FRCV, em especial na infância e adolescência, diferentes marcadores inflamatórios envolvidos em cada etapa do processo de formação da placa de ateroma têm sido estudados, entre eles os leucócitos.^5^ O número de leucócitos é reconhecido como um marcador inflamatório e um preditor de eventos cardiovasculares.^6^ Subpopulações de leucócitos estão relacionados ao processo inflamatório.^7^

Conhecendo a influência das condições de nascimento no desenvolvimento das DCV, objetivou-se avaliar a relação do número de leucócitos com os riscos cardiovasculares associados às características de nascimento, estado nutricional, composição corporal e exames bioquímicos.

## Método

Estudo transversal, desenvolvido com adolescentes de ambos os sexos, nascidos em Viçosa (MG), entre 1992 a 2001. A amostra foi distribuída conforme as fases da adolescência, assim descritas: inicial - 10 a 13 anos; intermediária - 14 a 16 anos; e tardia - 17 a 19 anos.^8^ O projeto foi aprovado pelo Comitê de Ética e Pesquisa com Seres Humanos da UFV (Processo n° 163/2012).

Para seleção da amostra usaram-se dados do Sistema de Informações sobre Nascidos Vivos (Sinasc) por meio do Datasus – Tecnologia de Informação a Serviço do SUS, filtrados pelo local de residência da mãe. Foi feita média do número de nascimento devido à subnotificação dos registros no sistema, totalizando 12.090 nascimentos referentes ao período da pesquisa. Esse total de nascimentos foi usado como população para definir o tamanho amostral pelo programa Epi Info, versão 6.04, a partir de fórmula específica para estudos transversais. Por se tratar de múltiplos FRCV como desfecho, usaram-se prevalência de 50%, que confere maior tamanho amostral, prevalência conservadora,^9^ variabilidade aceitável de 5% e nível de confiança de 95%. A amostra mínima totalizou 372 adolescentes. Devido à perda amostral esperada em função do intervalo de tempo da pesquisa e à possibilidade de não encontrar os dados de nascimento, acrescentaram-se 20% ao cálculo inicial e chegou-se a 447 indivíduos. Participaram do estudo 475 adolescentes.

A partir dos prontuários maternos de todos os partos hospitalares, construiu-se o banco de dados composto pelas variáveis de nascimento: peso, comprimento, perímetro cefálico e torácico, índice de Apgar e idade gestacional. O peso de nascimento foi classificado como baixo (<2.500g), insuficiente (2.500-2.999g), adequado (3.000-3.999g) e grande para a idade gestacional (>4.000g).^10^ O comprimento ao nascer e o perímetro cefálico foram classificados com os critérios do Ministério da Saúde e tiveram percentis como referência.^11^

A etapa seguinte constituiu-se em encontrar a população na fase da adolescência. A fonte de busca foram as escolas rurais e urbanas, públicas e privadas. Os indivíduos foram incluídos no estudo a partir da manifestação de interesse e identificação dos dados de nascimento, independentemente da fase da adolescência e com respeito à proporcionalidade por sexo. Caso contrário, esses eram excluídos e fazia-se novo sorteio entre os interessados. Após assinatura do Termo de Consentimento Livre Esclarecido (TCLE) pelos responsáveis ou pelos próprios adolescentes maiores de 18 anos, os sujeitos da pesquisa foram orientados sobre os cuidados prévios e dias para feitura dos exames bioquímicos, bioimpedância elétrica, exame clínico e avaliação nutricional.

Os dados antropométricos (peso, estatura) e de composição corporal e os bioquímicos foram coletados na Divisão de Saúde da Universidade Federal de Viçosa (MG). Posteriormente agendou-se retorno para aplicar os questionários socioeconômicos e de estilo de vida, além de entregar os resultados dos exames e avaliação nutricional.

Avaliou-se o peso em balança digital eletrônica Kratos^*®*^, capacidade máxima de 150kg, sensibilidade de 50g. Para medida da estatura, usou-se estadiômetro portátil Alturexata^*®*^, com extensão de 2,13m; resolução de 0,1cm. O índice de massa corporal (IMC) foi calculado pela razão entre peso corporal (kg) e estatura (m^2^), classificados em Escore-z, de acordo com sexo e idade, com o uso da proposta da Organização Mundial da Saúde.^12^ Para avaliar a composição corporal, usou-se a bioimpedância elétrica vertical com oito eletrodos táteis InBody 230^®^. O perímetro da cintura (PC) foi aferido no ponto médio entre a margem inferior da última costela e a crista ilíaca, em duplicata, com fita métrica flexível inelástica, com extensão de 2m, dividida em centímetros e subdivida em milímetros. Após essas avaliações, foi feito o cálculo da relação cintura-estatura (RCE) e do índice de adiposidade corporal (IAC).

Na avaliação bioquímica, dosaram-se colesterol total, lipoproteína de alta e baixa densidade (HDL e LDL) e triglicerídeos. A classificação do perfil lipídico baseou-se na I Diretriz de Prevenção de Aterosclerose na Infância,^13^ que considera como inadequados valores limítrofes e elevados. Essa referência também foi usada para avaliar a insulinemia de jejum e a resistência insulínica, essa calculada pelo modelo matemático Homeostasis Model Assessment – Insulin Resistance (HOMA-IR). A glicemia de jejum foi avaliada segundo o Comitê Internacional de Diagnóstico de *diabetes mellitus*, que considera glicemia de jejum alterada ≥100mg/dL. O hemograma completo e o ácido úrico foram classificados de acordo com o sexo e idade, conforme valores de referência do kit Bioclin-Quibasa (Quibasa Química Básica Ltda., Belo Horizonte, Brasil).

A contagem do número de leucócitos foi feita pelo método de impedância elétrica (aparelho Coulter T890), com o kit Beckman Coulter (Beckman Coulter GmbH, Krafeld, Alemanha) e valores de referência para até 12 anos (4.500-13.500mm^3^), 13 a 16 anos (4.500-13.000mm^3^), 17 a 18 anos (4.500-12.500mm^3^) e acima de 18 anos (4.500-11.500mm^3^).

Aferiu-se pressão arterial com base no protocolo estabelecido pela VI Diretriz Brasileira de Hipertensão Arterial,^14^ com monitor de pressão sanguínea de insuflação automática Omron^®^ Model HEM-741 CINT (Omron Healthcare Inc., Lake Forest, IL, USA), preconizado pela Sociedade Brasileira de Cardiologia, classificada a partir do P90.

Para o comportamento sedentário, avaliou-se o tempo parado na semana e no fim de semana em frente à televisão, videogame e computador, caracterizado como tempo de tela (TT), como critério de classificação considerou-se o TT ≥ 2 horas/dia.^15^

Para análise dos dados selecionou-se o Statistical Package for the Social Sciences (SPSS, IBM^®^, Chicago, IL, EUA) versão 20.0. Para determinar a normalidade das variáveis numéricas, usou-se o teste de Kolmogorov-Smirnov. Para comparar as variáveis numéricas, usaram-se os testes de Kruskal-Wallis para três ou mais grupos independentes, e o de Mann-Whitney, para dois grupos independentes.

A diferença entre as proporções foi avaliada pelos testes qui-quadrado e exato de Fisher quando necessário. Os modelos de regressão linear foram construídos com o Data Analysis and Statistical Software (Stata-Stata Corp., College Station, TX, EUA). Para a feitura das análises de regressão, as variáveis foram usadas na forma contínua. Para avaliar a associação entre número de leucócitos e fatores de risco CV, foi feito ajuste para sexo e fase da adolescência. As variáveis independentes incluídas nos modelos de regressão apresentaram *p*<0,20 na regressão simples. Para a análise dos modelos, a variável dependente, leucócitos, foi transformada em logaritmo. Devido ao fato de as variáveis antropométricas e de composição corporal apresentarem multicolinearidade, foram gerados cinco modelos diferentes de regressão múltipla. O nível de significância adotado foi α<0,05.

## Resultados

Participaram do estudo 475 adolescentes, 238 (50,1%) do sexo feminino. Na estratificação pela fase da adolescência, estudaram-se 221 (46,5%) na fase inicial, 132 (27,8%) na intermediária e 122 (25,7%) na final.

Muitos prontuários não apresentaram registros dos dados, como índice de Apgar e idade gestacional, principalmente nos anos iniciais da pesquisa. Porém, observou-se que a maior parte dos adolescentes apresentou índice de Apgar≥7 no primeiro e quinto minutos, 318 (94,6%) e 379 (98,9%) respectivamente, que implicam boa vitalidade fetal. Quanto à idade gestacional, observou-se que 97,8% dos nascimentos foram a termo (37-41 semanas), seis (1,9%) prematuros(≤36 semanas) e um (0,3%) pós-termo. Em relação ao tipo de parto, observaram-se 157 (33,4%) nascimentos por parto vaginal e 313 (66,6%) por cesariana.

A mediana de peso ao nascer foi de 3.100 g, maior entre os meninos (*p*<0,001), assim como o comprimento ao nascer (*p* = 0,007) e o perímetro cefálico (*p*<0,001) e torácico (*p* = 0,001). Na adolescência, observou-se que as adolescentes apresentaram maiores valores de %GC (*p*<0,001); PC (0,02); RCE (*p*<0,001) e IAC (*p*<0,001) e não houve diferença em relação ao IMC. O maior TT foi observado entre as adolescentes, em relação ao sexo masculino, sem diferença significativa (*p* = 0,09) (tabela 1).

**Tabela 1:** Mediana, valores mínimos e máximos das variáveis de nascimento e adolescência na amostra total e estratificado pelo sexo

		Amstar total			Masculino			Feminino		
		n (%)	Mediana (Mín‐Máx)		n (%)	Mediana (Mín‐Máx)		n (%)	Mediana (Mín‐Máx)	*p* ‐valor
*Nascimento*										
	Peso (g) a	474	3100 (1480‐4500)		236 (96,6)	3200 (1900‐4500)		238 (100)	3050 (1480‐4350)	<0,001 b
	Comprimento (cm) a	466	50 (38‐56)		234 (98,7)	50 (41‐56)		232 (97,5)	49 (38‐53)	0,007 b
	P Cef (cm) a	471	34 (28‐39)		235 (99,2)	34,5 (31‐39)		236 (99,2)	34 (28‐37)	<0,001 b
	PT (cm) a	470	33 (27‐39)		234 (98,7)	33 (29‐39)		236 (99,2)	33 (27‐37)	0,001 b
*Adolescência*										
	IMC (kg/m^2^)	475	19,46 (13,22‐40,05)		237 (100)	19,27 (13,22‐40,05)		238 (100)	19,67 (14,04‐34,90)	0,10 b
	%GC (%)	475	21 (5,6‐48,7)		237 (100)	16,1 (5,6‐43,5)		238 (100)	25,5 (9,1‐48,7)	<0,001 b
	P Cint (cm)	475	71 (51,2‐118)		237 (100)	69,5 (51,2‐118)		238 (100)	71,5 (53,5‐100)	0,02 b
	RCE (cm)	475	0,44 (0,35‐0,71)		237 (100)	0,43 (0,35‐0,67)		238 (100)	0,45 (0,36‐0,71)	<0,001 b
	IAC (%)	475	24,42 (14,19‐42,75)		237 (100)	22,07 (14,19‐41,25)		238 (100)	25,93 (18,46‐42,75)	<0,001 b
	Tempo tela (TT)/horas	475	128,57 (34,29‐240)		237 (100)	125,71 (42,86‐239,29)		238 (100)	132,99 (34,29‐240)	0,09 b

*Comp. sedentário*										
	TT ≥2h	274 (57,7)	–		108 (45,6)	–		93 (39,1)	–	0,15 c
	TT <2h	201 (42,3)	–		129 (54,4)	–		145 (60,9)	–	

a Falta registro nos prontuários maternos.

b Teste Mann‐Whitney.

c Qui‐quadrado.

Min, mínimo; Max, máximo; Comp., Comportamento; IMC, Índice de Massa Corporal; %GC, Percentual de Gordura Corpora; RCE, Relação Cintura‐estatura; IAC, Índice de Adiposidade Corporal; TT, Tempo de Tela; P Cef, perímetro cefálico; PT, perímetro torácico; P Cint, perímetro da cintura.

Neste estudo, não houve diferença entre número de leucócitos e peso de nascimento na amostra total (*p* = 0,92) ou estratificada pelo sexo, masculino (*p* = 0,77) ou feminino (*p* = 0,43). O mesmo aconteceu na avaliação das fases da adolescência (*p* = 0,42). O número de leucócitos foi maior nas adolescentes, quando comparadas com o sexo masculino (*p*<0,001) (tabela 2).

**Tabela 2:** Mediana, valores mínimo e máximo do número de leucócitos em relação às variáveis de nascimento, antropometria, exames bioquímicos e clínicos em adolescentes

		Leucócitos		
		n (%)	Mediana (Q1‐Q3)	*p* ‐valor
*Peso nascimento (g)*				
	<2500	35 (7,4)	5900 (4900‐6900)	0,92 *
	2500‐2999	121 (25,5)	5600 (4500‐6850)	
	3000‐3999	308 (64,8)	5700 (4900‐6800)	
	≥4000	10 (2,1)	5750 (4250‐6575)	

*Comprimento ao nascer (cm)*				
	<45	7 (1,5)	5900 (4800‐6300)	0,97 *
	≥45 a <53	446 (93,9)	5700 (4800‐6800)	
	≥53	22 (4,6)	5900 (5000‐6400)	

*Apgar 1° minuto*				
	≤6	18 (5,4)	6050 (5350‐7025)	0,13 **
	≥7	318 (94,6)	5600 (4675‐6700)	

*Apgar 5° minuto*				
	≤6	4 (1,1)	5450 (4200‐8725)	0,97 **
	≥7	279 (98,9)	5600 (4800‐6700)	

*Perímetro cefálico (cm)*				
	<32	19 (4,0)	6300 (5800‐6900)	0,30 *
	≥32 a <36	361 (76,0)	5700 (4800‐6800)	
	≥36	95 (20,0)	5500 (4700‐6600)	

*Fase da adolescência*				
	Inicial	221 (46,5)	5600 (4700‐6700)	0,42 *
	Intermediária	132 (27,8)	5700 (4800‐6975)	
	Final	122 (25,7)	5900 (4900‐6925)	

*Sexo*				
	Masculino	237 (49,9)	5400 (4600‐6350)	<0,001 **
	Feminino	238 (50,1)	6000 (5000‐7300)	

*Estado nutricional*				
	Baixo peso a ^‐^ b	21 (4,4)	5000 (4250‐6350)	
	Eutrófico a	345 (72,6)	5600 (4700‐6700)	0,008 *
	Excesso de peso b	109 (22,9)	6000 (5100‐7450)	

*Percentual gordura corporal (%)*				
	Eutrófico	275 (57,9)	5600 (4600‐6500)	0,002 **
	Excesso de gordura	200 (42,1)	6000 (4925‐7300)	

*IAC (%)*				
	Normal	424 (89,3)	5600 (4700‐6700)	0,002 **
	Alterada	51 (10,7)	6400 (5200‐7700)	

*Perímetro da cintura (cm)*				
	Normal	430 (90,5)	5600 (4700‐6700)	<0,001 **
	Alterada	45 (9,5)	6300 (5400‐8000)	
*RCE (cm)*				
	Normal	419 (88,2)	5600 (4700‐6700)	<0,001 **
	Alterada	56 (11,8)	6350 (5400‐7950)	

*Colesterol total (mg/dL)*				
	<150md/dL	196 (41,3)	5700 (4800‐6700)	0,93 **
	<150md/dL	279 (58,7)	5600 (4800‐6800)	

*Triglicerídeos (mg/dL)*				
	<100md/dL	408 (85,9)	5600 (4700‐6700)	0,001 **
	≥100md/dL	67 (14,1)	6300 (5300‐7400)	
*LDL (mg/dL)*				
	<100md/dL	310 (65,3)	5800 (4800‐7000)	0,13 **
	≥100md/dL	165 (34,7)	5500 (4850‐6500)	

*HDL (mg/dL)*				
	<45md/dL	307 (64,6)	5600 (4700‐6800)	0,18 **
	≥45md/dL	168 (35,4)	5900 (4925‐6775)	

*Comportamento sedentário*				
	TT≥2h	201 (42,3)	5690 (4900‐6800)	0,96 **
	TT<2h	274 (57,7)	5700 (4600‐6800)	

*Pressão arterial (mmHg)*				
	<P90	460 (96,8)	5700 (4800‐6800)	0,91 **
	≥P90	15 (3,2)	5800 (5400‐6200)	

* Teste Kruskal‐Wallis.

** Teste Mann‐Whitney.

a,b Letras iguais significam ausência de diferença estatística.

IAC, Índice de Adiposidade Corporal; RCE, Relação Cintura‐Estatura; TT, Tempo de tela; LDL, Lipoproteína baixa densidade; HDL, Lipoproteína de alta densidade; Q1, Primeiro Quartil; Q3, Terceiro Quartil

Na avaliação dos leucócitos na adolescência foram encontrados maiores valores nos indivíduos com excesso de peso (*p* = 0,004) e de gordura corporal (*p* = 0,02); IAC acima do P90 (*p* = 0,002); maiores valores de RCE (*p*<0,001) e PC (*p*<0,001).

Quanto ao perfil lipídico, apenas os triglicerídeos apresentaram diferença entre o número e leucócitos. Nos adolescentes com TG elevados os leucócitos eram mais elevados (*p* = 0,001).

Não foram encontradas diferenças estatísticas na contagem de leucócitos em relação ao comportamento sedentário e entre os adolescentes com pressão arterial maior do que P90.

Após análise de regressão linear simples entre variáveis de nascimento, bioquímicas, clínicas e antropométricas em relação ao número de leucócitos, os modelos finais ajustados para sexo e fase da adolescência são mostrados na tabela 3. Incluíram-se nesses modelos os eosinófilos, linfócitos, monócitos e plaquetas (*p*<0,001); triglicerídeos (*p* = 0,016); índice HOMA (*p* = 0,01); insulina (*p* = 0,005); perímetro cefálico (*p* = 0,14); perímetro torácico (*p* = 0,16); IMC (*p* = 0,0005); %GC (*p* = 0,001); RCE (*p*<0,001); PC (*p* = 0,004) e IAC (*p*<0,001). Os cinco modelos de regressão múltipla foram feitos a fim de verificar maior poder explicativo, porém observou-se que qualquer um dos modelos usados mostrou associação de parâmetros antropométricos e de composição corporal com a contagem de leucócitos. Independentemente da variável antropométrica usada para avaliar o número de leucócitos na adolescência em relação às variáveis supracitadas, com exceção do IAC e do %GC, todas apresentaram no modelo final a mesma associação com a fase da adolescência, sexo, número de plaquetas, eosinófilos, monócitos e linfócitos. Os modelos 01 (IMC) e 05 (PC) apresentaram os melhores coeficientes de determinação (R^2^ = 0,46). Após análise dos gráficos de resíduos para os cinco modelos, verificou-se que os resíduos distribuíram-se linearmente ao longo dos valores. Isso mostra que os modelos de regressão linear foram adequados. Observou-se também, nos gráficos de resíduos padronizados e valores preditos, que os primeiros distribuíram-se de modo uniforme em torno da média.

**Tabela 3:** Modelos finais de regressão linear de associação entre leucócitos, variáveis antropométricas, clínicas e laboratoriais ajustada por sexo e fase da adolescência

Variáveis	Modelo 1‐IMC (R^2^ = 0,46)				Modelo 2‐IAC (R^2^ = 0,45)				Modelo 3‐%GC (R^2^ = 0,44)		
	β	SE	IC95%		β	SE	IC95%		β	SE	IC95%
IMC (kg/m^2^)	0,010	0,002	0,005‐0,015		–	–	–		–	–	–
%GC (%)	–	–	–		–	–	–		0,004	0,001	0,002‐0,006
IAC (%)	–	–	–		0,009	0,002	0,004‐0,013		–	–	–
PC (cm)	–	–	–		–	–	–		–	–	–
RCE (cm)	–	–	–		–	–	–		–	–	–
F. Adol.	0,06	0,012	0,039‐0,088		0,08	0,012	0,058‐0,106		0,086	0,012	0,062‐0,109
Sexo	0,06	0,018	0,027‐0,100		–	–	–		–	–	–
Plaquetas (mil/mm^3^)	0,0005	0,0001	0,0002‐0,0009		0,0006	0,0001	0,0002‐0,0009		0,0005	0,0001	0,0002–0,0009
Eosinófilos (mm^3^)	0,0002	0,00003	0,0001‐0,0002		0,0001	0,00003	0,0001‐0,0002		0,0001	0,00003	0,0001–0,0002
Monócitos (mm^3^)	0,0004	0,00008	0,0003‐0,0006		0,0005	0,00008	0,0003‐0,0006		0,0005	0,00008	0,0003–0,0006
Linfócitos (mm^3^)	0,0002	0,00001	0,0001‐0,0002		0,0002	0,00001	0,0001‐0,0002		0,0002	0,00001	0,0001–0,0002

Variáveis	Modelo 4‐RCE (R^2^ = 0,45)							Modelo 5‐PC (R^2^ = 0,46)			
	β		SE		IC95%			β		SE	IC95%
IMC (kg/m^2^)	–		–		–			–		–	–
%GC (%)	–		–		–			–		–	–
IAC (%)	–		–		–			–		–	–
PC (cm)	–		–		–			0,004		0,0009	0,002–0,005
RCE (cm)	0,56		0,16		0,245–0,887			–		–	–
F. Adol.	0,08		0,012		0,056–0,104			0,06		0,012	0,038–0,088
Sexo	0,05		0,019		0,016–0,091			0,06		0,018	0,026–0,099
Plaquetas (mil/mm^3^)	0,0005		0,0001		0,0002–0,0008			0,0005		0,0001	0,0002–0,0009
Eosinófilos (mm^3^)	0,0001		0,00003		0,0001–0,0002			0,0002		0,00003	0,0001–0,0002
Monócitos (mm^3^)	0,0004		0,00008		0,0003–0,0006			0,0004		0,00008	0,0003–0,0006
Linfócitos (mm^3^)	0,0002		0,00001		0,0001–0,0002			0,0002		0,00001	0,0001–0,0002

IMC, índice de massa corporal; %GC, percentual de gordura corporal; IAC, índice de adiposidade corporal; PC, perímetro da cintura; RCE, relação cintura‐estatura; F. Adol., fase da adolescência; –, variável não incluída no modelo de regressão.

## Discussão

O presente estudo avaliou o comportamento de variáveis de nascimentos, antropometria, exames bioquímicos e clínicos na adolescência com o aumento do número de leucócitos, como marcador biológico para os riscos de DCV.

O estudo abordou adolescentes com valores de leucócitos dentro dos parâmetros de normalidade, uma vez que se objetivou avaliar a relação dessa variável com os fatores de risco CV. Em relação ao peso ao nascer não foram encontradas associações, porém, na adolescência, pode-se observar que indivíduos com maiores percentuais de gordura, excesso de peso e hipertrigliceridemia apresentaram maiores contagens desse marcador biológico para inflamação.

A avaliação da contagem de leucócitos relaciona-se a uma inflamação subclínica, não necessariamente é preciso encontrar valores alterados para se observar efeito ao longo do tempo. No processo inflamatório ocorre a ativação leucocitária, uma vez ativada inicia-se a produção de outros marcadores inflamatórios.

As condições de nascimento, em especial o peso ao nascer, são um fator relacionado ao desenvolvimento de DCV^1^ e podem estar vinculadas aos leucócitos, marcadores de risco biológico para essas doenças. Esse fato não pôde ser observado neste estudo, no qual o peso ao nascer não se associou às alterações no leucograma, diferentemente do estudo de base populacional feito no norte da Finlândia que mostrou associação de baixo peso ao nascer com aumento do número de leucócitos, principalmente nas adolescentes do sexo feminino. A identificação de associação de inflamação em adolescentes pode proporcionar prevenção de DCV na idade adulta.^16^

Neste estudo, não foram encontradas diferenças nos números de leucócitos em relação às fases da adolescência. Contrariamente a esse achado, altos níveis de inflamação se associaram aos leucócitos em adolescente de 13 a 16 anos que participaram de estudo na Inglaterra e no País de Gales.^17^

Maiores números de leucócitos foram observados no sexo feminino, achado que vem ao encontro do trabalho feito com adolescentes finlandeses e americanos, no qual as meninas apresentaram maiores números de leucócitos.^16^^,^ 18 O valor desse achado está relacionado à maior exposição do sexo feminino ao processo inflamatório devido às consequências fisiopatológicas desse estado, principalmente quando associado ao uso de contraceptivos orais, por ser esse considerado um risco independente para inflamação sistêmica.^16^

Os adolescentes do estudo com alteração nas variáveis antropométricas e de composição corporal apresentaram maiores números de leucócitos. Estudo feito em Alegre/Espírito Santo/Brasil também mostrou maiores médias de leucócitos e linfócitos para os adolescentes com maiores valores de PC e %GC.^19^ A obesidade é caracterizada por uma inflamação crônica que proporciona alterações no sistema imunológico e pode estar associada a *diabetes mellitus* tipo 2 e DCV.^20^ Nesse contexto, os leucócitos podem servir como biomarcador ou mesmo mediador e ligar obesidade, inflamação e resistência à insulina.^6^

Em relação ao perfil lipídico, apenas os adolescentes com triglicerídeos aumentados apresentaram diferença entre o número de leucócitos. Conhecendo a associação de triglicerídeos com o risco de aumento de doenças coronarianas^21^ e que os leucócitos podem ser considerados marcadores biológicos para DCV^16^, esses podem ser usados na prática clínica de atenção à saúde dos adolescentes no que tange à identificação dos riscos cardiovasculares.

A não associação, neste estudo, do número de leucócitos aos fatores de risco CV, como colesterol e LDL elevados, HDL baixo, comportamento sedentário e pressão arterial >P90, pode estar relacionada à homogeneidade da amostra em relação a essas variáveis; porém os dados encontrados assemelham-se ao Ten Towns Heart Health Study, que também não mostrou associação entre leucócitos e fatores tradicionais de risco cardiovascular. A relevância da identificação de marcadores de inflamação no início da vida está relacionada ao fato de essa desempenhar papel causal de associação, na fase adulta, entre leucócitos e DCV.^17^

As plaquetas relacionaram-se ao excesso de peso. O volume plaquetário médio é considerado um indicador da atividade plaquetária, seu aumento foi demonstrado em diversos eventos vasculares agudos e está relacionado com a obesidade.^22^ O aumento da ativação das plaquetas é conhecido por desencadear a aterosclerose e desempenha papel importante na sua progressão. A relação plaquetas e população de leucócitos está associada à maior frequência de desfechos cardiovasculares adversos.^23^ As células brancas do sangue estão relacionadas à aterosclerose independentemente dos fatores de risco e podem ser consideradas marcador de baixo custo, de fácil interpretação no diagnóstico da aterosclerose.^24^

A elevação da concentração total de células brancas é fator de risco independentemente de morbidade e mortalidade por doença coronariana, doença arterial periférica e acidente vascular cerebral.^25^ Estudo espanhol mostrou associação entre leucócitos e subpopulações com hipertrigliceridemia e HDL baixo, assim como para os componentes da síndrome metabólica. Indivíduos nos maiores quartis de leucócitos apresentavam risco aumentado de desenvolver a síndrome (*p*<0,001). Essa associação também foi observada para todos os subtipos de leucócitos, com exceção de basófilos.^26^ Esses dados corroboram os achados desse estudo no qual as subpopulações de leucócitos mantiveram-se em todos os modelos finais de regressão.

Dentre as subpopulações leucocitárias, os monócitos são descritos como o tipo celular predominante no perfil inflamatório em processos ateroscleróticos. Em indivíduos adultos com alterações endócrinas, os monócitos podem ser preditores de complicações macrovasculares.^27^

Os eosinófilos são os leucócitos multifuncionais envolvidos na iniciação e propagação de respostas inflamatórias e, portanto, têm papel na patogênese de doenças inflamatórias.^28^ Os eosinófilos têm um composto que se liga à molécula-1 de adesão da célula vascular (VCAM-1) no endotélio e se infiltram, após situar-se na região infectada ou inflamada, e fazem a fagocitose de pequenas partículas intermediadas pelo complexo antígeno-anticorpo.^28^

Os linfócitos mostram-se presentes na resposta imunológica em todas as fases do processo aterosclerótico e se relacionam a marcadores inflamatórios, quando analisados em relação a adolescentes obesos.^29^ Indivíduos com excesso de peso e de gordura corporal apresentam, além dos macrófagos infiltrados no tecido, a presença de linfócitos, que produzem citocinas e contribuem positivamente para a inflamação local do tecido.^30^

Pode-se concluir que, neste estudo, as variáveis de nascimento não se associaram às alterações nos números de leucócitos, enquanto as alterações antropométricas na adolescência mostraram-se boas indicadoras de maiores números de leucócitos, independentemente da fase da adolescência e sexo. Essas variáveis foram preditoras para o aumento do número de plaquetas e subpopulações de leucócitos.

Apesar de não ter sido encontrada neste estudo associação entre leucócitos e fatores de riscos cardiovasculares, como comportamento sedentário, pressão arterial >P90, colesterol e LDL elevados, HDL baixo, os leucócitos e suas subpopulações podem ser considerados biomarcadores efetivos e de baixo custo para identificação do risco cardiovascular em adolescentes, uma vez que se trata de uma inflamação subclínica. Assim, a avaliação do número de leucócitos pode ser mais um exame usado na prática clínica, pois se associa ao processo da aterogênese, na presença de fatores de risco CV e outros marcadores.

Este estudo apresenta limitações devido ao seu próprio desenho transversal, que impossibilita identificar e/ou interpretar a temporalidade das associações mostradas nos resultados. Porém, os resultados obtidos nesta pesquisa podem ser usados em outros estudos devido às características próprias, como o tamanho amostral, a representatividade da população de adolescentes no município e a similaridade desse grupo com os adolescentes brasileiros.

Conhecendo a relação da alteração dos valores de leucócitos com as alterações cardiovasculares e sabendo que tais processos se iniciam na infância e adolescência e persistem durante a fase adulta, é fundamental identificar esses adolescentes, com o objetivo de reduzir a exposição aos fatores de risco CV.
